# A prospective, randomized, double-blind, and multicenter trial of prophylactic effects of ramosetronon postoperative nausea and vomiting (PONV) after craniotomy: comparison with ondansetron

**DOI:** 10.1186/1471-2253-14-63

**Published:** 2014-08-04

**Authors:** Jung-Hee Ryu, Ji-Eun Lee, Young-Jin Lim, Deok-Man Hong, Hee-Pyoung Park, Jong-In Han, Hee-Jung Baik, Hyun-Zu Kim, Kyeong-Tae Min, Sang-Hwan Do

**Affiliations:** 1 Department of Anesthesiology and Pain Medicine, Seoul National University Bundang Hospital, 166 Gumi-ro, Bundang-gu, Seongnam-si, Gyeonggi-do 463-707, South Korea; 2 Department of Anesthesiology and Pain Medicine, Seoul National University Hospital, Seoul, South Korea; 3 Department of Anesthesiology and Pain Medicine, College of Medicine, Seoul National University, Seoul, South Korea; 4 Department of Anesthesiology and Pain Medicine, School of Medicine, Ewha Womans University, Seoul, Korea; 5 Department of Anesthesiology and Pain Medicine, Severance Hospital, Seoul, South Korea

**Keywords:** Craniotomy, Ondansetron, Postoperative nausea and vomiting, Ramosetron

## Abstract

**Background:**

Craniotomy patients have a high incidence of postoperative nausea and vomiting (PONV). This prospective, randomized, double-blind, multi-center study was performed to evaluate the efficacy of prophylactic ramosetron in preventing PONV compared with ondansetron after elective craniotomy in adult patients.

**Methods:**

A total of 160 American Society of Anesthesiologists physical status I–II patients aged 19–65 years who were scheduled to undergo elective craniotomy for various intracranial lesions were enrolled in this study. All patients received total intravenous anesthesia (TIVA) with propofol and remifentanil. Patients were randomly allocated into three groups to receive ondansetron (4 mg; group A, *n*  =  55), ondansetron (8 mg; group B, *n*  =  54), or ramosetron (0.3 mg; group C, *n*  =  51) intravenously at the time of dural closure. The incidence of PONV, the need for rescue antiemetics, pain score, patient-controlled analgesia (PCA) consumption, and adverse events were recorded 48 h postoperatively.

**Results:**

Among the initial 160 patients, 127 completed the study and were included in the final analysis. The incidences of PONV were lower (nausea, 14% vs. 59% and 41%, respectively; *P*  <  0.001; vomiting, *P*  =  0.048) and the incidence of complete response was higher (83% vs. 37% and 59%, respectively; *P*  <  0.001) in group C than in groups A and B at 48 h postoperatively. There were no significant differences in the incidence of PONV or need for rescue antiemetics 0–2 h postoperatively, but significant differences were observed in the incidence of PONV and complete response among the three groups 2–48 h postoperatively. No statistically significant intergroup differences were observed in postoperative pain, PCA consumption, or adverse events.

**Conclusion:**

Intravenous administration of ramosetron at 0.3 mg reduced the incidence of PONV and rescue antiemetic requirement in craniotomy patients. Ramosetron at 0.3 mg was more effective than ondansetron at 4 or 8 mg for preventing PONV in adult craniotomy patients.

**Trial registration:**

Clinical Research Information Service (CRiS) Identifier: KCT0000320. Registered 9 January 2012.

## Background

Postoperative nausea and vomiting (PONV) is one of the most common perioperative concerns, with a relatively high incidence of up to 73% after craniotomy [1-5]. PONV usually leads to patient discomfort, delayed discharge from the intensive care unit (ICU) or hospital, and increased medical costs [6]. Moreover, severe vomiting may result in dehydration, electrolyte imbalance, and acid–base disturbance [6].

Neurosurgical patients with PONV are at an increased risk of aspiration, intracranial or cerebral hypertension, hematoma formation, and neurological deterioration [7]. After craniotomy, PONV occurs with incidences up to 60% for emesis, and 70% for nausea when no prophylactic antiemetic is administered [8]. The etiology of PONV in patients undergoing craniotomy is probably multifactorial, and well-known risk factors of PONV include age, gender, a history of motion sickness, and previous PONV [9]. The effects of neurosurgical risk factors such as side and site of lesion, presence of midline shift and mass effect, and tumor pathology on PONV were evaluated, but no significant correlations were observed [3,10]. Fabling *et al*. [11] reported that PONV occurred frequently during the initial 48 h after craniotomy.

Various antiemetics, including anticholinergics and dopamine receptor antagonists, have been studied with regard to their efficacy for the prevention and treatment of PONV [1,5,12]. However, these agents have been reported to have adverse effects such as excessive sedation, hypotension, dysphoria, hallucinations, and extrapyramidal signs [1,5,12,13], all of which are undesirable for neurological assessment after neurosurgical operations. Selective 5-HT_3_ receptor antagonists (ondansetron, granisetron, and tropisetron) are effective in PONV after craniotomy without neurological adverse effects [1,3-5,7,11,14-17]. Ramosetron, a selective 5-HT_3_ receptor antagonist, has been used effectively in various surgical procedures for the prevention or treatment of PONV [18]. However, limited data are available regarding the efficacy of ramosetron for the prevention of PONV in neurosurgical patients and it was suggested that prophylactic administration of ramosetron may reduce the incidence of PONV after craniotomy compared with ondansetron. This prospective, randomized, double-blind, multi-center study was designed to evaluate the efficacy and safety profile of prophylactic administration of ramosetron for PONV in comparison with ondansetron after craniotomy.

## Methods

### Patients

The present study was performed at Seoul National University Hospital, Seoul National University Bundang Hospital, Severance Hospital, and Ewha Womans University Medical Center. The protocol was approved by the Ethics Committees of each of the above hospitals and registered in the Clinical Research Information Service (CRiS, KCT0000320). After obtaining written and informed consent, 160 adult patients (American Society of Anesthesiologists [ASA] physical status I or II, aged 19–65 years) undergoing elective craniotomy from January 2012 until March 2013 were included.

Exclusion criteria were gastric disease, pregnancy, a history of craniotomy, anticancer chemotherapy, antiemetic use within 24 h, severe renal (serum creatinine > 1.6 mg/dl) or hepatic insufficiency (liver enzymes more than twice the normal value), borderline QTc prolongation (>430 msec for males, > 450 msec for females) on electrocardiography, chronic use of opioids for more than 2 weeks, antidepressant medication, having undergone an emergency operation, or those who could not understand the numerical rating scale (NRS) or communicate (scheduled to be sedated postoperatively).

### Anesthesia

Anesthesia and monitoring were standardized for all patients. Patients received no preanesthetic medication. Standard monitoring included electrocardiography, pulse oximetry, and noninvasive blood pressure (NIBP) monitoring. Induction of anesthesia consisted of propofol (4 μg/ml) and remifentanil (3–4 ng/ml) using target controlled infusion (TCI). Neuromuscular blockade was performed using intravenous rocuronium 0.6 mg/kg to facilitate tracheal intubation. During anesthetic induction, a 20-gauge arterial catheter was inserted into a radial artery for continuous blood pressure (BP) monitoring or arterial blood sampling, and the subclavian vein was catheterized using a real-time ultrasound device to monitor central venous pressures. Propofol (2–4 μg/ml) and remifentanil (2–4 ng/ml) in oxygen and medical air (FiO_2_ 0.5) were used during maintenance of anesthesia. Ventilation was mechanically controlled to achieve end-tidal CO_2_ between 30 and 35 mmHg. Muscle relaxants were used as needed to maintain a single twitch on train-of-four stimulation. For intraoperative neurophysiological monitoring, no additional neuromuscular blocker was administered during surgery. Temperature was monitored using an esophageal stethoscope with thermistor and maintained at 36 ± 1°C with a warm pad throughout surgery. At the end of the surgery, an intravenous patient-controlled analgesia (PCA) device was connected; the PCA consisted of fentanyl 15 μg/ml (total, 100 ml) and was programmed to run with a 1-ml bolus dose and 10 min lockout time.

### Randomization and intervention

Before induction of anesthesia, the anesthesiologist responsible for patient allocation randomized the patients using a computer-generated random number table (Random Allocation Software Version 1.0) with block size 3. Allocation was concealed with numbered sealed envelopes. Patients were allocated randomly to one of three groups to receive 4 mg of ondansetron (group A), 8 mg ondansetron (group B), or 0.3 mg ramosetron (group C) intravenously. Identical 5-ml syringes containing the same volume (mixed with normal saline to the total volume of 4 ml) of ondansetron or ramosetron were prepared and administered at the end of surgery (dura mater closure) by blinded nurses to the group allocation.

### Outcomes

After the operation, the patients were transferred to the intensive care unit (ICU). All patients were observed for 48 h postoperatively. Every episode of nausea or vomiting was recorded under three assessment time frames, 0–2 h, 2–24 h, and 24–48 h, by a blinded nurse. Nausea was defined as a subjectively unpleasant sensation associated with the awareness of the urge to vomit; retching was defined as labored, spasmodic, rhythmic contraction of the respiratory muscles without expulsion of gastric contents; vomiting was defined as the forceful expulsion of gastric contents from the mouth [4,13]. Complete response was regarded as no PONV. The severity of nausea (NRS with 0 = none to 100 = most severe) was evaluated verbally to guide the use of rescue antiemetics and, therefore, is not presented as a result. The rescue antiemetic used was 10-mg metoclopramide administered intravenously. This was given to patients whose NRS for nausea was greater than 30 and for patients who experienced more than one episode of vomiting or wanted to be treated.

Postoperative pain scores (using NRS with 0 = none to 100 = most severe) and consumption of PCA were also assessed. All adverse events were reviewed and judged by the investigator, and the details of clinically significant adverse events were supposed to be submitted to the Ethics Committees. Other postoperative adverse events, such as drowsiness, dizziness, and QTc prolongation, were also recorded.

### Statistical analysis

In the previous study of Kathirvel *et al*., [3] the incidence of PONV 24 h postoperatively was 44% in patients with administration of ondansetron 4 mg after craniotomy. A reduction of 30% in PONV with ramosetron was considered clinically significant. The analysis showed that 46 patients per group would be sufficient to detect the antiemetic effect of ramosetron (α = 0.0175 and β = 0.2). We chose 55 patients per group assuming a 20% drop-out rate.

Continuous variables (age, height, weight, duration of surgery and anesthesia, pain score, and PCA consumption) were analyzed by ANOVA, and categorical variables (gender, ASA physical class, type of operation, preoperative dexamethasone, incidence of PONV and complete response, rescue analgesics, and adverse events) were compared using the chi-squared test or Fisher’s exact test. *Post hoc* comparisons were made with Student Newman Keuls test. Data are expressed as means ± standard deviation (SD) or counts (%). A *p* value less than 0.05 was deemed to indicate statistical significance.

## Results

**Figure 1:**
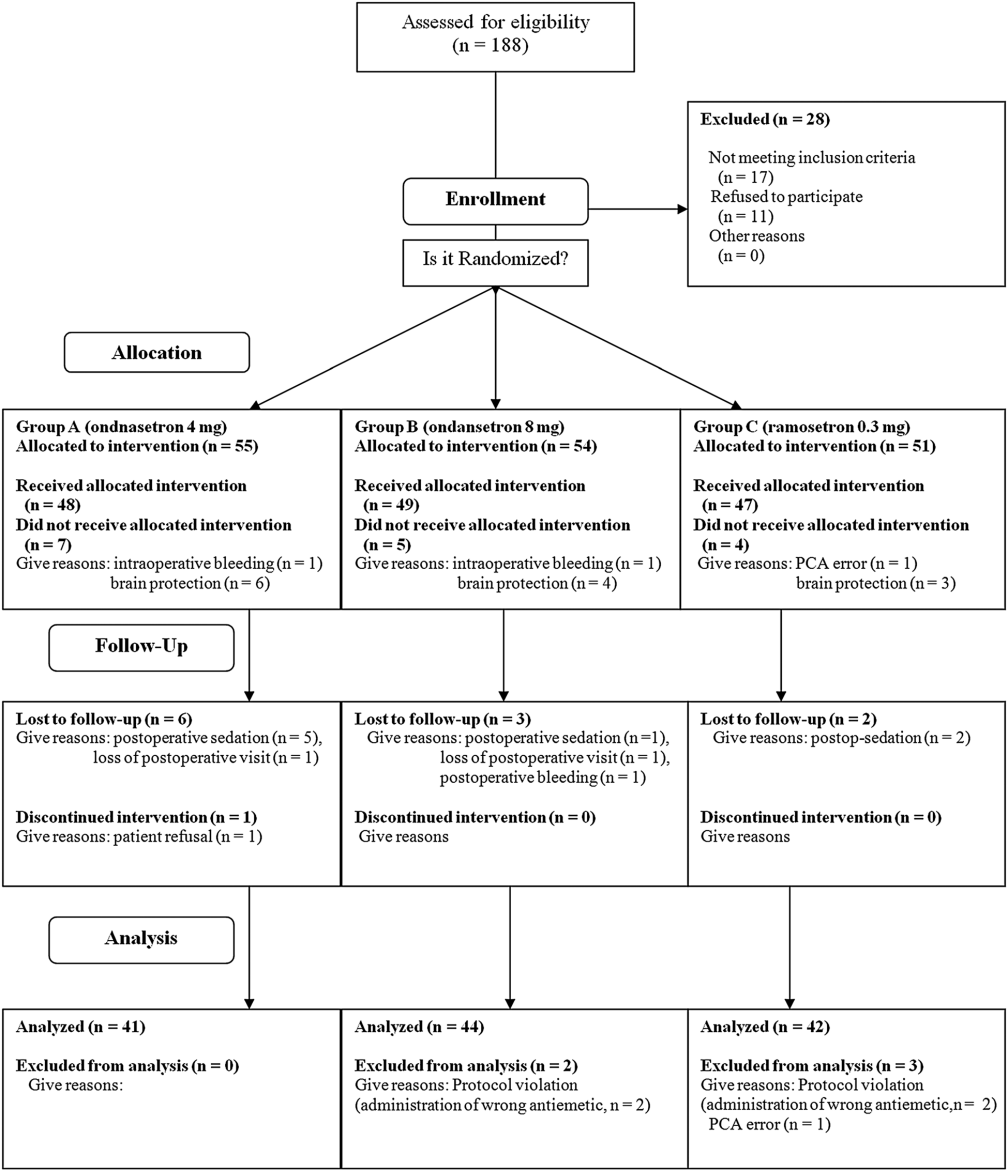
Flow diagram of the patients.

A total of 160 patients from four hospitals were enrolled in the present study, and 33 were excluded during allocation or the follow-up period. Ultimately, a total of 127 patients completed the study (Figure 1). Twenty-one patients were chosen to be sedated to protect their brains intra-operatively or postoperatively, and other patients were excluded due to perioperative massive bleeding (*n* = 3), lack of a postoperative patient visit (*n* = 2), minor protocol violations (*n* = 6; errors in the PCA regimen and incorrect rescue antiemetics), and patient refusal (Figure 1). These violations did not impact subject safety and were considered minor. Patient characteristics and information on surgery and anesthesia are summarized in Table 1, and there were no statistically significant differences among the three groups with respect to patient demographic data.

**Table 1:** Patients and surgery characteristics

	**Group A (n = 41)**	**Group B (n = 44)**	**Group C (n = 42)**	***P *** **value**
Age (yr)	49 (21–65)	48 (27–65)	53 (23–64)	0.677
Weight (kg)	62 ± 11	62 ± 11	63 ± 11	0.894
Height (cm)	162 ± 8	163 ± 8	163 ± 9	0.728
Gender (M/F)	14/27	19/25	13/29	0.471
Preoperative dexamethasone	17 (41)	15 (34)	11 (26)	0.339
Operation time (min)	249 ± 87	288 ± 117	254 ± 110	0.179
Anesthesia time (min)	321 ± 90	368 ± 131	327 ± 114	0.110
Type of surgery				0.322
Tumor surgery	26 (63)	26 (59)	20 (48)	
Vascular surgery	15 (37)	18 (41)	22 (52)	
ASA class I/II	24 (59)/17 (41)	22 (50)/22 (50)	22 (52)/20 (48)	0.720
Preexisting disease, (n)				
Hypertension	11	13	13	0.916
Diabetes mellitus	4	5	7	0.609
Liver disease	3	2	0	0.223

Group A; ondansetron 4 mg group. Group B; ondansetron 8 mg group. Group C; ramosetron 0.3 mg group. Values are expressed as mean (SD) or number of the patients (n) except age (mean, range).

The results for the 0- to 48-h study period suggested that the incidence of nausea (P < 0.001) and the need for rescue antiemetics (P = 0.008) were lower in group C than in groups A or B (Table 2). Additionally, the incidence of complete response (P < 0.001) was significantly higher in group C than in groups A and B (Table 2). However, statistically significant differences in the incidence of PONV or complete response were not observed between A and B groups throughout the study period.

**Table 2:** Incidences of PONV and rescue antiemetics

	**Group A (n = 41)**	**Group B (n = 44)**	**Group C (n = 42)**	***P *** **value**
Postoperative 0–48 h				
Nausea	24 (59)	18 (41)	6 (14) ^† ‡^	< 0.001
Retching & Vomiting	9 (22)	9 (20)	3 (7)	0.13
Complete response	15 (37)	26 (59)	35 (83) ^†^	< 0.001
Rescue antiemetic	11 (27)	9 (20)	1 (2) ^† ‡^	0.008
Postoperative 0–2 h				
Nausea	6 (15)	5 (11)	2 (5)	0.318
Retching & Vomiting	1 (2)	1 (2)	1 (5)	0.999
Complete response	34 (83)	40 (91)	40 (95)	0.172
Rescue antiemetic	2 (5)	3 (7)	0 (0)	0.249
Postoperative 2–24 h				
Nausea	17 (41)	12 (27)	3 (7) ^† ‡^	0.001
Retching & Vomiting	6 (15)	8 (18)	2 (2)	0.154
Complete response	23 (56)	33 (75)	38 (86) ^†^	0.002
Rescue antiemetic	8 (20)	5 (11)	1 (2) ^†^	0.045
Postoperative 24–48 h				
Nausea	16 (39)	14 (32)	4 (10) ^†^	0.006
Retching & Vomiting	4 (10)	5 (11)	0 (0)	0.088
Complete response	25 (61)	30 (68)	38 (90) ^†^	0.006
Rescue antiemetic	5 (12)	6 (14)	0 (0)	0.050

Group A; ondansetron 4 mg group. Group B; ondansetron 8 mg group. Group C; ramosetron 0.3 mg group. Values are expressed as number of the patients (%). ^†^: <0.0175 compared with Group A. ^‡^: 0.0175 compared with Group B.

There were no significant differences in the incidence of PONV and the need for rescue antiemetics 0–2 h postoperatively. However, significant differences were observed in the incidence of PONV and complete response among the three groups 2–48 h postoperatively (Table 2). Nausea occurred less frequently and the rate of complete response was higher in group C than in group A or B 2–24 and 24–48 h postoperatively (Table 2). Additionally, rescue antiemetics were less frequently required in group C than in groups A or B 2–48 h postoperatively (Table 2).

Total incidences of PONV for subgroups that did or did not receive dexamethasone were analyzed (Table 3). The incidence of nausea was lower and that of complete response was higher in group C than group A in both patients with or without steroid (Table 3).

**Table 3:** Incidences of PONV and rescue analgesic in patients with or without steroid

***Patients without steroid***	**Group A (n = 17)**	**Group B (n = 15)**	**Group C (n = 11)**	***P *** **value**
Nausea	10 (21)	6 (40)	0 (0)^†^	0.007
Retching & Vomiting	3 (17)	1 (7)	0 (0)	0.265
Complete response	7 (41)	9 (60)	11 (100)^†^	0.007
Rescue antiemetic	4 (24)	2 (13)	0 (0)	0.214
*Patients without steroid*	**Group A (n = 24)**	**Group B (n = 29)**	**Group C (n = 31)**	***P *** **value**
Nausea	14 (58)	12 (41)	6 (19)^†^	0.012
Retching & Vomiting	6 (25)	8 (27)	3 (10)	0.178
Complete response	9 (37)	17 (58)	24 (77)^†^	0.011
Rescue antiemetic	7 (29)	7 (24)	1 (3)^†^	0.025

Group A; ondansetron 4 mg group. Group B; ondansetron 8 mg group. Group C; ramosetron 0.3 mg group.

Values are expressed as number of the patients (%). †: < 0.0175 compared with Group A.

No significant inter-group difference was observed in terms of postoperative pain or PCA consumption (Table 4). Common adverse events related to ramosetron treatment are headache, dizziness, and drowsiness. In the present study, headache was not checked because craniotomy patients were enrolled. Rates of other adverse events, including drowsiness and dizziness, did not reach statistical significance among the groups (Table 4). There were no serious adverse events, such as QTc prolongation of electrocardiography or withdrawal of the study drugs.

**Table 4:** Postoperative pain, recovery profiles and postoperative adverse effects

	**Group A (n = 41)**	**Group B (n = 44)**	**Group C (n = 42)**	***P *** **value**
Postoperative 0–2 h				
Pain (VRS)	36 (0–80)	41 (0–90)	39 (0–100)	0.974
PCA consumption (ml)	1.5 (0–7)	1.5 (0–8)	1.5 (0–5)	0.921
Postoperative 2–24 h				
Pain (VRS)	27 (0–100)	26 (0–85)	26 (0–70)	0.792
PCA consumption (ml)	11 (0–46)	12 (20–75)	13 (0–78)	0.541
Postoperative 24–48 h				
Pain (VRS)	17 (0–80)	15 (0–60)	18(0–70)	0.589
PCA consumption (ml)	16 (1–95)	26 (0.2-100)	22 (0–88)	0.345
Advervse events				
Drowsiness	4 (10)	5 (11)	2 (5)	0.528
Dizziness	7 (17)	14 (32)	6 (14)	0.101
Itching	1 (2)	1 (2)	0 (0)	0.604
Others	1 (2)	0 (0)	0 (0)	0.347

Group A; ondansetron 4 mg group. Group B; ondansetron 8 mg group. Group C; ramosetron 0.3 mg group. Values are expressed as median (range) or number of the patients (%).

## Discussion

The present study was designed to evaluate the antiemetic efficacy of ramosetron in adult patients undergoing elective craniotomy. The overall incidence rates of PONV during the first 48 h after surgery were 63%, 41%, and 17% in the ondansetron 4 mg, ondansetron 8 mg, and ramosetron 0.3 mg groups, respectively. There was no placebo group in this study because high incidences of PONV after craniotomy were reported in previous investigations [1,3,4]. Administration of ramosetron 0.3 mg at the time of dural closure significantly reduced the incidence of PONV and the requirement of rescue antiemetics compared with ondansetron 4 mg or 8 mg 48 h postoperatively without significant adverse events.

Ondansetron, one of the most common 5-HT_3_ receptor antagonists, has been investigated for the prevention of PONV after craniotomy, and discrepant results have been reported regarding the effectiveness of ondansetron in neurosurgical patients [1,11,14,15,17,19,20]. Conversely, previous studies have indicated that the proper timing of ondansetron administration is at the time of dural closure for the prevention of PONV in neurosurgical patients [3,11,17]. Fabling *et al*. [11] recommended ondansetron 8 mg for the prevention of PONV in high-risk neurosurgical patients. In the present study, the incidences of PONV after prevention with ondansetron 4 and 8 mg were 63 and 41%, respectively.

Ramosetron, another selective 5-HT_3_ receptor antagonist, has been reported to be effective for the prevention and treatment of PONV after various surgeries [21-27]. Previous investigations have shown that ramosetron 0.3 mg was more effective than ondansetron 4 mg in patients with spine surgery, total knee arthroplasty, and laparoscopic cholecystectomy [22,24,27] and as effective as ondansetron 8 mg in patients with gynecological surgery and laparoscopic surgery [25,27]. However, no data have been reported concerning the antiemetic efficacy of ramosetron for preventing PONV after craniotomy. It is noteworthy that ramosetron 0.3 mg was more effective than ondansetron 8 mg after craniotomy in the present study. This may have been because the incidence and degree of PONV after craniotomy are higher than those in other surgeries.

Several factors should be taken into consideration during investigations of PONV in patients with craniotomy. PONV in neurosurgical patients is affected by postcraniotomy pain and PCA consumption [4]. In the current study, there were no statistically significant differences in postcraniotomy pain and PCA consumption among the groups, and these factors may be considered to have minimal effect on the results. Additionally, instead of inhalation agents, total intravenous anesthesia with propofol and remifentanil was used for maintenance of anesthesia to monitor the evoked potential and reduce PONV. The most frequent adverse event following administration of the selective 5-HT_3_ receptor antagonist is headache. Headache was not recorded because it is difficult to discriminate headache from postcraniotomy pain in patients with neurosurgery.

These phenomena could be explained by the potency of the two drugs. The results of previous meta-analyses showed that ramosetron was effective for preventing PONV without adverse effects and also had statistically significant differences for prevention of early and late PONV compared with ondansetron [18,28]. However, ramosetron (about US $23 for 0.3 mg) is more expensive than ondansetron (US $5 for 4 mg and US $8 for 8 mg) in Korea. The choice and use of antiemetics should be individualized considering cost effective benefits.

The present study had a few limitations. First, the study population consisted of patients with various intracranial diseases, including tumors and vascular lesions. Additionally, patients with intracranial tumors may be using preoperative dexamethasone to reduce cerebral edema. Dexamethasone, a corticosteroid, has effective antiemetic action and reduces the degree and incidence of PONV [29], although few studies have investigated the effects of preoperative steroid use on the incidence of PONV in neurosurgical patients. In the present study, no significant differences were found in the number of patients with preoperative steroid use among the three groups, a finding that may cause some (but minimal) confusion in the interpretation of the results. Second, the incidences of PONV in patients with craniotomy were reported to be high, and combined antiemetic measures with different sites of action are recommended for this high-risk group [9]. The aim of the present study was to evaluate the efficacy of ramosetron alone for the prevention of PONV in neurosurgical patients, and further studies of combination antiemetics for neurosurgical patients are needed. Third, the sample size calculation was based on the previous investigation of Kathirvel et al. and the incidence of PONV was 44% [3]. However, all patients of Kathirvel’s investigation received dexamethasone for 24 hours whereas 34% of the patients in this study were administered preoperative dexamethasone. In addition, anesthesia was maintained with N2O and isoflurane in the previous study of Kathirvel *et al*. [3] instead of TIVA using propofol-remifentanil of the present study. These factors may have influenced on the incidence of PONV. Forth, minimal calculated sample size estimated to detect the difference in the primary outcome among the three study groups (46 patients per group) was not finally achieved because more patients than we expected have been dropped out during study period of the 4 centers. This may influence the statistical significance in that the lack of difference between the two ondansetron groups could be related to inadequate sample size.

## Conclusion

Intravenous administration of ramosetron 0.3 mg at the end of surgery was more effective than ondansetron 4 or 8 mg for the prevention of PONV in patients undergoing elective craniotomy under total intravenous anesthesia using propofol and remifentanil. Future studies of multimodal prophylactic strategies with ramosetron and other antiemetics in this high-risk patient population are needed.

## Abbreviations

BP: Blood pressure; ICU: Intensive care unit; NIBP: Noninvasive blood pressure; NRS: Numerical rating scale; PCA: Patient-controlled analgesia; PONV: Postoperative nausea and vomiting; TCI: Target controlled infusion; TIVA: Total intravenous anesthesia.

## Competing interests

The authors have no conflicts of interest or financial ties to disclose.

## Authors’ contributions

JHR, YJL, HJP, KTM and SHD contributed study design. JEL, DMH, HPP, JIH, and HZK collected and analyzed data. JHR and SHD drafted the manuscript. JHR, HJB and KTM made critical revisions of the manuscript. All authors read and approved the final analysis of the manuscript.

## Pre-publication history

The pre-publication history for this paper can be accessed here:

http://www.biomedcentral.com/1471-2253/14/63/prepub

## References

[B1] Fabling JM Gan TJ El-Moalem HE Warner DS Borel CO A randomized, double-blinded comparison of ondansetron, droperidol, and placebo for prevention of postoperative nausea and vomiting after supratentorial craniotomy Anesth Analg 2000 91 2 358 361 10910848 10.1097/00000539-200008000-00023

[B2] Fabling JM Gan TJ Guy J Borel CO El-Moalem HE Warner DS Postoperative nausea and vomiting. A retrospective analysis in patients undergoing elective craniotomy J Neurosurg Anesthesiol 1997 9 4 308 312 9339401

[B3] Kathirvel S Dash HH Bhatia A Subramaniam B Prakash A Shenoy S Effect of prophylactic ondansetron on postoperative nausea and vomiting after elective craniotomy J Neurosurg Anesthesiol 2001 13 3 207 212 11426094 10.1097/00008506-200107000-00005

[B4] Madenoglu H Yildiz K Dogru K Kurtsoy A Guler G Boyaci A Randomized, double-blinded comparison of tropisetron and placebo for prevention of postoperative nausea and vomiting after supratentorial craniotomy J Neurosurg Anesthesiol 2003 15 2 82 86 12657991 10.1097/00008506-200304000-00003

[B5] Pugh SC Jones NC Barsoum LZ A comparison of prophylactic ondansetron and metoclopramide administration in patients undergoing major neurosurgical procedures Anaesthesia 1996 51 12 1162 1164 9038459 10.1111/j.1365-2044.1996.tb15060.x

[B6] Kovac AL Prevention and treatment of postoperative nausea and vomiting Drugs 2000 59 2 213 243 10730546 10.2165/00003495-200059020-00005

[B7] Neufeld SM Newburn-Cook CV The efficacy of 5-HT3 receptor antagonists for the prevention of postoperative nausea and vomiting after craniotomy: a meta-analysis J Neurosurg Anesthesiol 2007 19 1 10 17 17198095 10.1097/01.ana.0000211025.41797.fc

[B8] Eberhart LH Morin AM Kranke P Missaghi NB Durieux ME Himmelseher S Prevention and control of postoperative nausea and vomiting in post-craniotomy patients Best Pract Res Clin Anaesthesiol 2007 21 4 575 593 18286838 10.1016/j.bpa.2007.06.007

[B9] Gan TJ Meyer T Apfel CC Chung F Davis PJ Eubanks S Kovac A Philip BK Sessler DI Temo J Gan TJ Meyer T Apfel CC Chung F Davis PJ Eubanks S Kovac A Philip BK Sessler DI Temo J Tramèr MR Watcha M Consensus guidelines for managing postoperative nausea and vomiting Anesth Analg 2003 97 1 62 71 Table of contents 12818945 10.1213/01.ane.0000068580.00245.95

[B10] Irefin SA Schubert A Bloomfield EL DeBoer GE Mascha EJ Ebrahim ZY The effect of craniotomy location on postoperative pain and nausea J Anesth 2003 17 4 227 231 14625709 10.1007/s00540-003-0182-8

[B11] Fabling JM Gan TJ El-Moalem HE Warner DS Borel CO A randomized, double-blind comparison of ondansetron versus placebo for prevention of nausea and vomiting after infratentorial craniotomy J Neurosurg Anesthesiol 2002 14 2 102 107 11907389 10.1097/00008506-200204000-00003

[B12] Meng L Quinlan JJ Assessing risk factors for postoperative nausea and vomiting: a retrospective study in patients undergoing retromastoid craniectomy with microvascular decompression of cranial nerves J Neurosurg Anesthesiol 2006 18 4 235 239 17006120 10.1097/00008506-200610000-00003

[B13] Watcha MF White PF Postoperative nausea and vomiting. Its etiology, treatment, and prevention Anesthesiology 1992 77 1 162 184 1609990 10.1097/00000542-199207000-00023

[B14] Hartsell T Long D Kirsch JR The efficacy of postoperative ondansetron (Zofran) orally disintegrating tablets for preventing nausea and vomiting after acoustic neuroma surgery Anesth Analg 2005 101 5 1492 1496 16244017 10.1213/01.ANE.0000181007.01219.38

[B15] Jain V Mitra JK Rath GP Prabhakar H Bithal PK Dash HH A randomized, double-blinded comparison of ondansetron, granisetron, and placebo for prevention of postoperative nausea and vomiting after supratentorial craniotomy J Neurosurg Anesthesiol 2009 21 3 226 230 19543000 10.1097/ANA.0b013e3181a7beaa

[B16] Wang YJ Cheng ZG Guo QL Clinical observation of granisetron in preventing postoperative nausea and vomiting following supratentorial craniotomy Hunan Yi Ke Da Xue Xue Bao 2002 27 6 545 546 12658936

[B17] Wig J Chandrashekharappa KN Yaddanapudi LN Nakra D Mukherjee KK Effect of prophylactic ondansetron on postoperative nausea and vomiting in patients on preoperative steroids undergoing craniotomy for supratentorial tumors J Neurosurg Anesthesiol 2007 19 4 239 242 17893575 10.1097/ANA.0b013e3181557471

[B18] Kim WO Koo BN Kim YK Kil HK Ramosetron for the prevention of postoperative nausea and vomiting (PONV): a meta-analysis Korean J Anesthesiol 2011 61 5 405 412 22148090 10.4097/kjae.2011.61.5.405 PMC3229020

[B19] Habib AS Keifer JC Borel CO White WD Gan TJ A comparison of the combination of aprepitant and dexamethasone versus the combination of ondansetron and dexamethasone for the prevention of postoperative nausea and vomiting in patients undergoing craniotomy Anesth Analg 2011 112 4 813 818 21081776 10.1213/ANE.0b013e3181ff47e2

[B20] Sinha PK Tripathi M Ambesh SP Efficacy of ondansetron in prophylaxis of postoperative nausea and vomiting in patients following infratentorial surgery: a placebo-controlled prospective double-blind study J Neurosurg Anesthesiol 1999 11 1 6 10 9890379 10.1097/00008506-199901000-00002

[B21] Choi DK Chin JH Lee EH Lim OB Chung CH Ro YJ Choi IC Prophylactic control of post-operative nausea and vomiting using ondansetron and ramosetron after cardiac surgery Acta Anaesthesiol Scand 2010 54 8 962 969 20626355 10.1111/j.1399-6576.2010.02275.x

[B22] Choi YS Shim JK Ahn SH Kwak YL Efficacy comparison of ramosetron with ondansetron on preventing nausea and vomiting in high-risk patients following spine surgery with a single bolus of dexamethasone as an adjunct Korean J Anesthesiol 2012 62 6 543 547 22778890 10.4097/kjae.2012.62.6.543 PMC3384792

[B23] Choi YS Shim JK Yoon Do H Jeon DH Lee JY Kwak YL Effect of ramosetron on patient-controlled analgesia related nausea and vomiting after spine surgery in highly susceptible patients: comparison with ondansetron Spine (Phila Pa 1976) 2008 33 17 E602 E606 18670328 10.1097/BRS.0b013e31817c6bde

[B24] Hahm TS Ko JS Choi SJ Gwak MS Comparison of the prophylactic anti-emetic efficacy of ramosetron and ondansetron in patients at high-risk for postoperative nausea and vomiting after total knee replacement Anaesthesia 2010 65 5 500 504 20337618 10.1111/j.1365-2044.2010.06310.x

[B25] Kim SI Kim SC Baek YH Ok SY Kim SH Comparison of ramosetron with ondansetron for prevention of postoperative nausea and vomiting in patients undergoing gynaecological surgery Br J Anaesth 2009 103 4 549 553 19700442 10.1093/bja/aep209

[B26] Lee JW Park HJ Choi J Park SJ Kang H Kim EG Comparison of ramosetron’s and ondansetron’s preventive anti-emetic effects in highly susceptible patients undergoing abdominal hysterectomy Korean J Anesthesiol 2011 61 6 488 492 22220226 10.4097/kjae.2011.61.6.488 PMC3249571

[B27] Ryu J So YM Hwang J Do SH Ramosetron versus ondansetron for the prevention of postoperative nausea and vomiting after laparoscopic cholecystectomy Surg Endosc 2010 24 4 812 817 19707823 10.1007/s00464-009-0670-5

[B28] Mihara T Tojo K Uchimoto K Morita S Goto T Reevaluation of the effectiveness of ramosetron for preventing postoperative nausea and vomiting: a systematic review and meta-analysis Anesth Analg 2013 117 2 329 339 23757469 10.1213/ANE.0b013e31829847a1

[B29] De Oliveira GS Jr Castro-Alves LJ Ahmad S Kendall MC McCarthy RJ Dexamethasone to prevent postoperative nausea and vomiting: an updated meta-analysis of randomized controlled trials Anesth Analg 2013 116 1 58 74 23223115 10.1213/ANE.0b013e31826f0a0a

